# The Use of Antibiotics for the Prevention of Surgical Site Infections in Two Government Hospitals in Taif, Saudi Arabia: A Retrospective Study

**DOI:** 10.7759/cureus.26731

**Published:** 2022-07-11

**Authors:** Omar M Alsaeed, Abdullah A Bukhari, Adel A Alshehri, Faisal A Alsumairi, Anas M Alnami, Hatim A Elsheikh

**Affiliations:** 1 College of Medicine, Taif University, Taif, SAU; 2 Department of Pharmacology, College of Medicine, Taif University, Taif, SAU

**Keywords:** guidelines for prophylactic antibiotics, surgical site infections, prophylactic, preoperative, postoperative, length of hospital stay, antibiotics

## Abstract

Background

Surgical site infections (SSIs) are among the most common postoperative complications. To reduce the occurrence of surgical wound infections, suitable antimicrobials should be administered.

Aim of the work

This study investigated the prophylactic use of antibiotics to prevent SSIs, in terms of agents and/or combination preferences, and the adherence of surgeons to both national and international guidelines of surgical antibiotic prophylaxis in Taif city government hospitals.

Methods

This is a retrospective study using a chart review of patients' medical records in two government hospitals in Taif city, Saudi Arabia, from July 2016 to July 2018. While maintaining the confidentiality of the patient’s data, it was collected and analyzed using the chi-squared and Fisher’s exact tests, and the difference between means was tested using the student’s t-test.

Results

Prophylactic antibiotics were used in 157 patients who had surgery (Group 1) while 52 patients did not receive preoperative antibiotics (Group 2). The most commonly used prophylactic antibiotics were metronidazole, cefuroxime, cefazolin, and ceftriaxone. Other antimicrobials used included cefotaxime, amoxicillin/clavulanic acid, cephalexin, and amoxicillin. Surgical site infections developed in one patient of those who received antibiotics preoperatively and in three Group 2 patients. The mean hospital length of stay in Group 1 was 38.5±9.2 hours, and 57.3±12.1 hours in Group 2 patients. The types of antibiotics used were generally within the scope of national and international recommended guidelines.

Conclusion

The use of preoperative prophylactic antibiotics significantly reduces the development of surgical site infections and the mean length of hospital stay. Various antimicrobial drugs that were used prophylactically in surgical practice are within the recommendations of both national and international guidelines.

## Introduction

Surgical site infections (SSIs) are considered one of the most common and preventable health care problems [1] that correlate with high morbidity and mortality [2]. SSIs are associated with increased hospital and ICU readmission, long-term complications of the surgical site, and even death [3]. Previous studies identified many risk factors for SSIs in various types of surgeries such as diabetes, cigarette smoking, systemic steroid use, obesity, extremes of age, poor nutritional status, coincident remote site infections or *Staphylococcus aureus* nasal colonization, and perioperative transfusion of certain blood products. The type of antibiotic prophylaxis used is considered one of the most important methods to reduce the risk of SSIs [4-5]. Properly administered antimicrobials for prophylaxis reduce the occurrence of surgical site wound infections. The timing of antibiotics administration is one of the major factors that affect their efficacy. If an antibiotic is used for prophylaxis incorrectly, for example, due to wrong timing or overconsumption [6], this has been shown to increase the occurrence of the drug’s side effects [7], treatment costs [8], super-infections, and the growth of new strains of microorganisms resistant to the effect of antimicrobial agents [7,9].One of the main roles of preoperative antibiotics administration is to reduce the risk of postoperative wound site infections [10]. There is growing evidence that supports the routine use of antibiotic prophylaxis [11]. Prophylactic antibiotics have to be administered intravenously within 30 minutes to one hour prior to the skin incision to reach the maximum concentration during the procedure [12-16]. Antibiotics selection is influenced by several factors such as the procedure site and the expected encountered organism [17-18]. However, some studies show that the antibiotics most commonly used either pre or postoperatively are Cephalosporins (first, second, or third generation), such as cefazolin, cefuroxime, or ceftriaxone, and penicillin-type antibiotics, such as Augmentin, amoxicillin, and ampicillin [19-20]. According to the American Center for Disease Control and Prevention (CDC) guidelines, in the absence of infection, antibiotic prophylaxis should be stopped 24 hours after surgery [21]. The antibiotic dosage should be calculated according to body weight [22]. For efficient prevention of SSIs, prophylactic antimicrobials alone are not sufficient, so other measures must be considered such as the control of some risk factors, instrument sterilization, and proper skin preparation [23]. A study published in 2018 investigated the adherence of surgeons in King Faisal Specialist Hospital and Research Centre (KFSH&RC), Riyadh, to the international guidelines of antimicrobial prophylaxis, which concluded that there was a wide gap between international and national standards and actual practice [24]. Another study was conducted in Rabigh General Hospital, Jeddah, with the same objective, published in 2019. This study, on the contrary, concluded that the level of adherence to the guidelines was fairly high among most surgeons [25]. The Saudi National Antimicrobial Therapy Guidelines, 2018 edition, include a list of proposed antimicrobial agents to be used in different cases. For example, for uncomplicated appendectomies and laparoscopic procedures like cholecystectomies in adults, these are IV Cefazoline and IV metronidazole, cephalosporin, gentamicin, or vancomycin as alternative choices [26]. In addition, the American Society of Health-System Pharmacists (ASHP), 2013 guidelines, in association with the Infectious Diseases Society of America, Surgical Infection Society, and Society for Healthcare Epidemiology of America, also proposed a list of recommendations for the types of antibiotic agents to be used in each different surgical procedure [27]. The aim of this study was to evaluate the use of prophylactic antibiotics in the prevention of SSIs, in terms of agents and/or combination preferences, and to investigate the adherence of the surgeons in Taif city government hospitals to the national and international guidelines of surgical antibiotic prophylaxis.

## Materials and methods

In this retrospective chart review and database study, the patients were identified in the computerized hospital administrative registration system of two governmental hospitals in Taif city (King Faisal Medical Complex and King Abdulaziz Specialist Hospital), Saudi Arabia. The study was conducted after the attainment of ethical approval number 137 from the institutional review board (IRB) of the Research and Studies Department, Directorate of Health Affairs, Taif, Saudi Arabia (HAP-02-T-067). A total of 209 male and female patients met the inclusion criteria in the reviewed files for the period from July 2016 to July 2018. The inclusion criteria included patients who underwent clean-contaminated surgeries and whose files were complete and included the type of surgery, the type of prophylactic antibiotic used, postoperative follow-up data, and the length of hospital stay. The exclusion criterion was clean surgeries. These are uninfected operative wounds in which no inflammation is encountered, and the respiratory, alimentary, genital, or uninfected urinary tract is not entered. In addition, clean wounds are primarily closed and, if necessary, drained with closed drainage. Operative incisional wounds that followed no penetration or blunt trauma were included if they met the criteria, as were children (14 years and less) and extremes of age (70 years and above).

Statistical analysis was carried out using SPSS (IBM SPSS Statistics for Windows, version 22.0, IBM Corp., Armonk, NY, USA). Group differences were analyzed using the chi-squared test and Fisher’s exact test, and differences between the means were tested using the student’s t-test. The level of significance was determined at P < 0.05.

## Results

The total number of patients enrolled in this study was 209, 89 of whom were male and 120 were female. They were divided into two groups according to whether they received prophylactic antibiotics preoperatively (Group 1), which included 157 patients, or had not received prophylactic antibiotics (Group 2), which were 52 patients (Table 1).

**Table 1:** Demographic data and types of surgeries performed according to the administration of antibiotics in patients prophylactically (Group 1) or not (Group 2) ٭ Statistically significant difference at the level of P ≤ 0.05 1 The mean age of both males and females for both groups is calculated along with the standard deviation (mean age ± SD).

Variable	Prophylactic Group (Group 1)	Non-Prophylactic Group (Group 2)
Total number of patients	157	52
Males	60	29
Females	97	23
Mean age of males (years)^1^	28.4±6.9	28.4±6.2
Mean age of females (years)^1^	36.6±8.8	37.3±7.1
Laparoscopic cholecystectomy	71	17
Open appendectomy	71	32
Laparoscopic appendectomy	15	3
Surgical wound infection	1/157 (0.6%)	3/52 (5.8%) ٭

Group 1 included 60 males and 97 females. The types of surgical procedures that were performed in Group 1 patients included 71 laparoscopic cholecystectomies, 71 open appendectomies, and 15 laparoscopic appendectomies. Group 2 included 52 patients who never received prophylactic and were not on preoperative antibiotics, of whom 32 had open appendectomies, three had laparoscopic appendectomies, and 17 had laparoscopic cholecystectomies. Surgical site infection developed in one out of 157 patients in Group 1 (0.6%), which is significantly lower than the number reported in three out of 52 patients (5.8%) in Group 2 (P≤ 0.05) (Table 1).

Table 2 shows the antibiotic agents most commonly used prophylactically. They were either given alone or in combination with another antibiotic. The most commonly used were metronidazole in 53 cases, cefuroxime in 50 cases, cefazolin in 22 cases, and ceftriaxone in 19 cases. Other antimicrobials included cefotaxime, amoxicillin/clavulanic acid, cephalexin, and amoxicillin. The doses were determined empirically.

**Table 2:** The prophylactic antibiotics used alone or in combination in the 157 patients of Group 1 *The antibiotic was used alone.

	Metronidazole	Cefuroxime	Cefazolin	Ceftriaxone	Cefotaxime	Amoxicillin/Clavulanic Acid	Cephalexin	Amoxicillin	Number of Patients
Metronidazole	3*	28	5	10	3	2	1	1	53
Cefuroxime	28	20*	2	-	-	-	-	-	50
Cefazolin	5	2	14*	-	-	-	-	-	22
Ceftriaxone	10	-	-	9*	-	-	-	-	19
Cefotaxime	3	-	-	-	4*	-	-	-	7
Amoxicillin/ clavulanic acid	2	-	-	-	-	1*	-	-	3
Cephalexin	1	-	-	-	-	-	1*	-	2
Amoxicillin	1	-	-	-	-	-	-	-	1
Number of Patients	53	50	22	19	7	3	2	1	157

Table 3 shows the mean hospital stay for each type of surgery according to whether the patients received prophylactic antibiotics or not, regardless of the type of antibiotic regimen they were on. In all types of surgeries, the length of stay was reported as significantly shorter (P≤0.05) in patients who had preoperative prophylaxis. Moreover, in patients who had a laparoscopic cholecystectomy, the combination of metronidazole and cephazolin appeared to have the best effect corresponding with a length of stay of 31.3±5.9 hours. Those who underwent open appendectomy had the best results with the combination of metronidazole and ceftriaxone at 35.4±6.6 hours. The patients who underwent a laparoscopic appendectomy showed better results with the combination of metronidazole and cefuroxime with a length of stay of 28.9±5.4 hours. The total mean hospital stay in Group 1 was 38±9 hours, which was significantly shorter than the reported 57±12 hours in Group 2 (P-value = 0.034). The most commonly used were metronidazole in 53 cases, cefuroxime in 50 cases, cefazolin in 22 cases, and ceftriaxone in 19 cases. Other antimicrobials included cefotaxime, amoxicillin/clavulanic acid, cephalexin, and amoxicillin. All agents were administered intravenously.

**Table 3:** Mean hospital length of stay (in hours) according to the type of surgery and prophylactic use of antibiotics The values in the table are means ± SD. ٭ significantly different from Group 1 value at the level of P≤ 0.05 1 Mean hospital length of stay for all 157 patients of Group 1, who received preoperative prophylactic antibiotics. 2 Mean hospital length of stay for all 52 patients of Group 2, who had not received preoperative prophylactic antibiotics.

Type of Surgery	Group 1	Group 2
Laparoscopic cholecystectomy	46.7±11.5	51.9±11.1 ٭
Open appendectomy	37.4±7.4	60.8±14.9 ٭
Laparoscopic appendectomy	29.8±7.6	47.7 ±9.2 ٭
All surgeries	38.5±9.2^1^	57.3±12.1 ٭^2^

## Discussion

The number of patients who underwent surgery after being administered prophylactic antibiotics (Group 1) was about 75% (157 out of 209 patients). This number shows that surgeons in the two hospitals were aware of the importance of prophylactic antibiotics in surgery for reducing the incidence of infection and length of hospital stay. The present study shows that the rate of infection in the prophylactic antibiotic group was lower than that in the group of patients who did not receive antibiotics prior to surgery. Furthermore, the shortest hospital stay was detected when metronidazole was combined with other antibiotics, in particular with cefazolin in laparoscopic cholecystectomy and with ceftriaxone in open appendectomies. Similar results were obtained previously by Sadraei-Moosavi and his co-workers [28], and Matsui and his colleagues [29], where the latter concluded, in their study, for a reappraisal of previously reported meta-analyses on antibiotic prophylaxis in low-risk laparoscopic cholecystectomies that prophylactic antibiotics reduce the incidence of postoperative infections and postoperative hospital stay. Preoperative antibiotics are administered prophylactically before surgery to reduce the risk of postoperative wound site infections [10]. There is growing evidence that supports the routine use of preoperative antibiotic prophylaxis, especially in procedures requiring excessive dissection or the use of an artificial implant as in hernia repairs or orthopedic or vascular surgeries [10-11,13]. Recent comparative studies proved that prophylactic antibiotics reduce the risk of wound infection in over 80% of orthopedic replacement surgeries [11,13,16]. In agreement with the findings of this study, previous studies proved that the best route of administration in such conditions is the intravenous route [10,16-18]. Prophylactic antibiotics have to be administered within 30 minutes to one hour prior to skin incision to reach the maximum concentration during the procedure; however, certain antibiotics, such as levofloxacin, clindamycin, and vancomycin, have to be given one hour earlier due to longer infusion administration time [12,17,21]. To prevent SSIs, antibiotic selection must consider several factors such as procedure site and the expected encountered organism. It was found that SSIs are commonly caused by *Staphylococcus aureus* or *epidermidis*, *beta-hemolytic streptococci*, *anerobic cocci*, and *Cutibacterium acne* (the latter isolated in certain orthopedic procedures, such as shoulder surgery) [14-15]. In the present study, all the antibiotics used were bactericidal, and their spectrum was broad. Ideally, the selected prophylactic antibiotic has a narrow spectrum, is effective against the expected causative organism, has an effective concentration in the surgical site, is safe, of suitable cost, and can be easily administered. Furthermore, in prophylaxis, bactericidal antimicrobials should be used, as the immune system is not properly active [18]. Cefazolin and cefoxitin are the most frequently used antibiotics to prevent SSIs except with drug-related allergies (alternatives: clindamycin or gentamycin) or a *methicillin-resistant Staphylococcus aureus *infection (alternative: Vancomycin), and when the expected organism is not susceptible to these drugs, as in lower gastrointestinal surgery (metronidazole is usually added) [23-24]. Our findings are that the most commonly used antibiotic is metronidazole, followed by cefuroxime and cefazolin. This is in agreement with ASHP recommendations for prophylactic agent use, the Saudi National Antimicrobial Therapy Guidelines for Community and Hospital Acquired Infections, 2018 edition, and the study by Tolba et al. on the use of prophylactic antibiotics to prevent SSIs in Saudi Arabia [24,26-27]. An extra dose must be given one hour before surgery in patients receiving preoperative antibiotics for another infection suiting the operative circumstances, except in patients with impaired kidney function and when using vancomycin, in which case, cefazolin or cefoxitin should be used [23]. Another dose is required in most circumstances dependent on many factors such as antibiotic half-life, the length of the procedure, expected dilution due to blood loss, and fluid replacement [30-31]. If a tourniquet is used, a reduced effect is expected, and special precautions in timing would be considered [17]. According to CDC guidelines, in the absence of infection, antibiotic prophylaxis should be stopped 24 hours after surgery except in some cardiothoracic procedures and in clean and clean-contaminated wounds, when no antimicrobial prophylaxis should be administered following the closure of the surgical incision [21]. The limitation of the duration of antibiotic use is mandatory to decrease the risk of the development of resistant organisms and alteration of the patient and/or hospital flora [10,14]. Antibiotics dosages should be calculated according to body weight, especially in children (gentamycin: 5 mg/kg; vancomycin: 15 mg/kg), even though some authors prefer empirical adult doses regardless of their weight for certain antibiotics such as cefazolin or cefoxitin (2 g) and ertapenem (1 g) [10,15,21]. In this report, the dose was empirically determined. In splenectomy patients, polyvalent vaccination against *pneumococci*, *Hemophilus type B*, and *meningococcus group C* should be considered [14]. For efficient prevention of SSIs, prophylactic antimicrobials alone are not sufficient, and other measures must be considered to control some risk factors, such as diabetes and renal impairment, for example, instrument sterilization and proper skin preparation [10,17,31]. According to Murray and his colleagues, a combination of soap-and-water/chlorohexidine wash of the operative site would reduce the *coagulase-negative Staphylococcus* and *Corynebacterium *colony count threefold compared to non-combined wash [32]. Patients with positive *Methicillin-resistant Staphylococcus aureus* cultures taken from their nostrils can be locally treated with 2% mupirocin or 5% povidone-iodine solution in addition to systemic vancomycin [33]. To conclude, there are some limitations to this study that can be noted. First of all, the sample size could have been increased by extending the study period for more than two years and by including more hospitals in Taif city in the study. This could have given more insight into antibiotics use during surgery in order to increase the accuracy of the results and to provide a wider scope of the clinical practice in various health care centers. Second, the study was limited to the adult age group. Including younger patients would have enriched the data about the clinical practice of surgical wound infection treatment in this subset of patients. Third, the types of surgical procedures chosen in the study were limited to open appendectomy and cholecystectomy, both open and laparoscopic. Finally, missing data in the medical records, precisely those related to the occurrence of adverse effects of antibiotics used (if any), in addition to missing laboratory results in several cases, such as the white blood cell count and inflammatory markers, hindered the inclusion of valuable data related to the use of antibiotics in the treatment of surgical wound infection in the study.

## Conclusions

In conclusion, the use of prophylactic antibiotics in the surgical practice of the two hospitals in this study is shown to be practiced successfully for preventing surgical wound infections and shortening the length of the hospital stay. The most commonly used prophylactic antibiotics were found to be metronidazole, cefuroxime, cefazolin, and ceftriaxone. In the two Taif city hospitals, the use of preoperative prophylactic antibiotics was shown to be in accordance with national and international guidelines.
